# Implementation strategies to scale up self-administered depot medroxyprogesterone acetate subcutaneous injectable contraception: a scoping review

**DOI:** 10.1186/s13643-023-02216-2

**Published:** 2023-07-04

**Authors:** Adeniyi Kolade Aderoba, Petrus Schoken Steyn, James Njogu Kiarie

**Affiliations:** 1 Centre for Tropical Medicine and Global Health, Nuffield Department of Medicine, University of Oxford, Oxford, UK; 2 Centre for Population Health and Interdisciplinary Research, Box 603, HealthMATE 360, Ondo Town, Ondo State 350001 Nigeria; 3 UNDP/UNFPA, UNICEF/WHO/World Bank Special Programme of Research, Development and Research Training in Human Reproduction, World Health Organization Headquarters, Geneva, Switzerland

**Keywords:** Implementation strategies, Scale-up, Self-care, Subcutaneous depot medroxyprogesterone acetate, Injectable contraception, Scoping review

## Abstract

**Background:**

Self-administered depot medroxyprogesterone acetate subcutaneous injectable contraception (DMPA-SC) is registered in many countries. It shows great potential for improving contraceptive access, continuation, and autonomy. However, there are challenges in rolling out this new efficacious intervention, and major implementation problems have been encountered during scale-up.

**Objective:**

To describe the implementation strategies to scale up self-administered DMPA-SC and the barriers, facilitators, and outcomes of these programs.

**Method:**

Recent guidelines, including the Preferred Reporting Items for Systematic Reviews and Meta-Analyses (PRISMA) extension for scoping reviews, were used to design and report this review. An article or report was eligible for inclusion if it reported interventions that could scale up self-administered DMPA-SC implementation or its facilitators, barriers, or outcomes. We searched six electronic databases and the grey literature for eligible articles and reports. Two reviewers independently screened the document titles, abstracts, and full texts to identify eligible documents. Data were extracted using structured forms. Using the Effective Practice and Organization of Care (EPOC) taxonomy of health systems framework for thematic analysis, data were presented in a narrative approach.

**Results:**

Of the 755 retrieved documents, 34 were included in this review. Most of the documents included were multi-country reports (*n* = 14), and all documents were published within the last 5 years (2018–2021). This review identified documents that reported interventions in all EPOC domains. The most-reported interventions were: task-sharing amongst health workforce cadres, engaged leadership, encouraging policies, training and education, DMPA-SC demand generation, integration into existing programs, improved funding mechanisms, collaboration with development partners, and supply chain strengthening. The main barriers were suboptimal funding, inadequate human resources, and poor logistics supply of DMPA-SC. There was minimal evidence of scale-up outcomes.

**Conclusion:**

This scoping review reported a wide range of interventions employed by countries and programs to scale up DMPA-SC self-administration but minimal evidence of the scale-up outcomes. Evidence from this review can help design better programs that improves access to quality family planning services to achieve the Sustainable Development Goals (SDG) targets 3.7. However, efforts should focus on rigorous implementation research that assess scaled up self-administered DMPA-SC interventions and report their outcomes.

**Registration:**

The protocol for this review was registered in the protocols.io repository (https://www.protocols.io/view/a-protocol-for-a-scoping-review-of-implementation-x54v9yemmg3e/v1).

**Supplementary Information:**

The online version contains supplementary material available at 10.1186/s13643-023-02216-2.

## Contributions to the literature

- Although self-administration of depot medroxyprogesterone acetate subcutaneous injectable contraception (DMPA-SC) is effective and safe, implementing and scaling up this intervention has been challenging.
- This scoping review identified multiple implementation strategies in all four domains of the Effective Practice and Organization of Care (EPOC) taxonomy of health systems framework that are deployed in DMPA-SC self-administration scale-up programs. Our results also show the need to improve the reporting of program scale-up outcomes using existing frameworks.
- Implementation strategies identified in this review can help design better scale up programs that improve access to quality family planning services to achieve the Sustainable Development Goals (SDG) targets 3.7.

## Introduction

Self-administered depot medroxyprogesterone acetate subcutaneous injectable contraception (DMPA-SC) is feasible, safe, and effective [1]. A recent meta-analysis of three randomized controlled trials found a significantly higher rate of 1-year continuation among women who self-administered DMPA-SC compared with those assigned to return to a provider for the injections [2]. These findings demonstrated the great potential of self-administration to improve contraceptive access, continuation, and autonomy, including in low-income and middle-income countries. Depot medroxyprogesterone acetate subcutaneous injectable contraception for self-injection has been registered in many countries [3, 4]. Self-administration of DMPA-SC takes place in both health facilities and the community [5–7]. However, there are challenges in rolling out this new efficacious intervention, and major implementation problems have been encountered during scale-up [8–13]. One challenge is identifying the barriers that prevent effective implementation and strategies to mitigate these problems [14]. Furthermore, many of these interventions are described in the grey literature and are not published in peer-reviewed journals, of which systematic reviews have been conducted.

### Objective

This study aimed to describe the implementation strategies to scale up self-administered DMPA-SC programs, the barriers and facilitators of these programs, and the outcomes of the implementation strategy.

## Methods

This scoping review was developed with Arksey and O'Malley’s approach [15], the updated methodological guidance for conducting a Joanna Briggs Institute scoping review [16], and the Preferred Reporting Items for Systematic reviews and Meta-Analyses (PRISMA) extension for scoping reviews [17]. Further guidance were from adapting guidelines for systematic review searches [18]. In line with the recommendations of Arksey and O'Malley [3], the methodological quality of the included documents in this review was not assessed. We registered the protocol for this review in the protocols.io repository (https://www.protocols.io/view/a-protocol-for-a-scoping-review-of-implementation-x54v9yemmg3e/v1).

### Inclusion criteria

#### Types of participants

Studies or reports involving women seeking contraception, contraceptive providers, and other relevant stakeholders were eligible.

#### Type of intervention

A peer-reviewed publication, journal article, project report, or other sources (hereafter referred to as “document”) was eligible if it reported interventions with the potential to scale-up self-administered DMPA-SC implementation programs or the barriers and facilitators to such programs. The World Health Organization (WHO) defines scaling up as “deliberate efforts to increase the impact of successfully tested health innovations to benefit more people and foster policy and program development on a lasting basis” [19]. Although pilot testing of self-administered DMPA-SC was exempted, programs starting with strategic planning for institutionalizing and expanding self-administered DMPA-SC were included.

Factors that enable or impede implementation scale-up were referred to as facilitators and barriers, respectively [20, 21]. Additionally, the outcomes of the implementation strategies were as defined by Proctor et. al. [22]. These include implementation outcomes (acceptability, adoption, appropriateness, costs, feasibility, fidelity, penetration, or sustainability), service outcomes (efficiency, safety, effectiveness, equity, patient-centeredness, or timeliness), and patient outcomes (satisfaction, function, or symptomatology).

#### Context

This scoping review considers all geographical locations and settings, and it was not limited by the date, language, or context. Observational or analytical quantitative, qualitative, or mixed method studies published in peer-reviewed journals or reports in the grey literature on interventions with the potential to scale-up self-administered DMPA-SC implementation programs or the barriers and facilitators of such programs were included. Abstracts with sufficient information were also included.

### Exclusion criteria

Studies were excluded if they focused entirely on (1) programs that pilot test or roll out DMPA-SC self-administration without a scale-up component, (2) implementation science theoretical and conceptual development, and (3) clinical treatment or adverse outcomes. Clinical trial protocols were excluded from this review. Additionally, editorials, opinion pieces, letters, guidelines, and review articles, including scoping and systematic reviews, were ineligible because our search strategy was designed to map DMPA-SC self-administration interventions from their source published articles and grey literature project reports.

### Information sources and search strategy

The following databases were searched: Cumulative Index to Nursing and Allied Health Literature (CINAHL; EBSCOhost), EMBASE (OvidSP), MEDLINE (OvidSP), Scopus (www. scopus. com), Google Scholar (https://scholar.google.com/), and Web of Science (core collection). Relevant thesaurus headings for “DMPA-SC” and “self-administration” were used, along with free-text search strings constructed for the title or abstract fields. The search for eligible studies was not limited by the date, language, or context, and the details of this process are provided as supplementary material.

Grey literature was identified by searching the following resources. First, we searched the websites of organizations, networks, and collaborations working on DMPA-SC implementation research, such as the DMPA-SC Access Collaborative Resource Library led by Program for Appropriate Technology in Health (PATH) in partnership with John Snow, Inc. (https://fpoptions.org/). Second, we conducted a Google search. Third, we sent requests using online networks and listservs for people implementing programs on DMPA-SC, such as the WHO IBP Network (https://ibpnetwork.org/) and CoreGroup-Reproductive, Maternal, Newborn, Child and Adolescent Health, and Health Systems Working Groups (https://coregroup.org/our-work/working-groups/#1502865240907-2c473617-a151).

The grey literature search used the keywords “DMPA-SC” and “self-administration” or their adaptations. The first 100 search hits were reviewed on websites with multiple pages of search results. Additionally, the reference lists of all eligible studies were manually searched for relevant documents. The search strategy was peer-reviewed using the Peer Review of Electronic Search Strategies (PRESS) guideline [23]. All database searches and requests for grey literature were conducted between October and December 2021. The full electronic search strategy is provided in the supplementary material. Search results from the different databases were merged in the Covidence systematic review application to facilitate deduplication, and data were chatted in Microsoft Excel.

### Data management

#### Selection of studies

After removing duplicates, the search results were first screened by their titles and abstracts for eligible studies using inclusion and exclusion criteria. The selected full-text documents were subjected to full eligibility screening. Reasons for exclusion at each screening stage were documented. Search results and included or excluded studies were summarized in a PRISMA flow diagram. Two independent reviewers screened and selected the documents. “Google Translate” was employed to screen titles and abstracts that are not in English, and advisers with appropriate language skills screened full text. Discrepancies were resolved by consensus between the reviewers or discussion with the scoping review team.

#### Data*** e***xtraction

Data were extracted from each study using a structured form developed and pretested for this purpose. Two independent reviewers extracted the data from each study. The information extracted included the following: (1) author(s), (2) year of publication, (3) journal or other types of documents, (4) time of data collection (years) or data sources, (5) country(ies), (6) objective of the study, (7) study design and analysis method, (8) targeted population(s), (9) interventions to scale up self-administered DMPA-SC implementation programs or the barriers and facilitators of such programs, (10) outcomes, recommendations, and lessons learned from interventions, and (11) any other relevant extraction topic. Discrepancies were resolved by consensus between the reviewers or discussion with the scoping review team.

#### Data analysis and synthesis

A narrative approach and “document counting” of the number of documents reporting an intervention to scale-up DMPA-SC self-administration were employed. The characteristics of the included studies, such as author(s) and year of publication, timeframe, study design and setting, country of study, characteristics of the study populations, implementation program framework, DMPA-SC intervention approach or strategies, barriers, and facilitators identified, and implementation outcomes, were summarized. A meta-analysis was not planned because this review aimed to describe the scope of interventions to scale up DMPA-SC programs and identify potential gaps and opportunities for improvement.

Thematic analysis of the different aspects of this scoping review was conducted using a priori frameworks. This involved:

- Mapping the DMPA-SC implementation strategy for each study with the Cochrane Effective Practice and Organization of Care (EPOC) taxonomy of the health system framework [24]. The EPOC taxonomy covers four health domains: healthcare delivery arrangements, financial arrangements, governance arrangements, and implementation strategies. Delivery arrangements refer to how, when, and where DMPA-SC client self-administration was organized and delivered, and who delivered DMPA-SC client self-administration activities [24]. The EPOC taxonomy financial arrangement denotes insurance schemes, how funds were collected, how services were purchased, and the use of targeted financial incentives or disincentives for DMPA-SC client self-administration [24]. We defined governance arrangements as rules or processes that affect how powers were exercised, particularly regarding authority, accountability, openness, participation, and coherence of DMPA-SC client self-administration programs [24]. Implementation strategies were interventions designed to bring about changes in healthcare organizations, the behavior of healthcare professionals, and the use of health services by healthcare recipients [24].
- Describing the outcome of scaling up DMPA-SC programs in terms of implementation, service, and client outcomes as defined by Proctor et al. [22].

If applicable, the absence of data in any theme was noted.

## Results

**Fig. 1:**
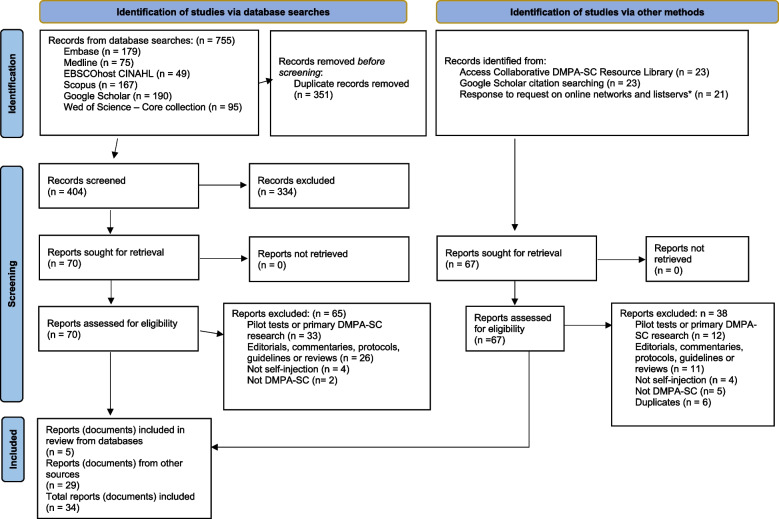
PRISMA flow diagram for the DMPA-SC Self-Administration Scoping Review

Figure 1 shows the PRISMA flow diagram for this scoping review. A total of 755 documents were retrieved from systematic searches of six databases. After deduplication, 404 document titles and abstracts were screened, and 334 ineligible documents were excluded. The full text of the remaining 70 documents was reviewed further, and five peer-reviewed articles were eligible for inclusion in this scoping review.

Sixty-seven documents were identified from other sources, 23, 23, and 21 were identified from the Access Collaborative DMPA-SC Resource Library, Google Scholar search for citations of included studies, and responses to requests on online networks and listservs, respectively. The 67 documents identified from other sources were retrieved and assessed for eligibility. Of these, 29 documents, including two peer-reviewed articles, met the inclusion criteria for this study. Therefore, 34 documents from six databases and other sources were included in this scoping review. The search results and a description of the selection process are shown in the PRISMA flow diagram in Fig. 1.

Table 1 summarizes the documents included in this review. Of the 34 documents, 7 (20.6%) were peer-reviewed journal articles, while 27 (79.4%) were reports, such as country briefs or project reports. Most of the documents included were multi-country reports (*n* = 14, 41.2%), and all were published within the last 5 years (2018–2021). Information from eligible studies was examined concerning the main categories of the EPOC taxonomy, namely, healthcare delivery arrangements, financial arrangements, governance arrangements, and intervention categories.

**Table 1:** Summary of included documents

**No.**	**Author and year of publication**	**Title**	**Document Type**	**Country**	**Project description**	*Intervention domain (s)
1	Burke et al. 2020 [25]	Adolescent and covert family planning users' experiences self-injecting contraception in Uganda and Malawi: implications for waste disposal of subcutaneous depot medroxyprogesterone acetate	Journal Article	Malawi and Uganda	DMPA SC self-injection scale-up program involving adolescents (15–19 years) and adults (20–49 years)	1, 3, 4
2	Katz et al. 2020 [26]	An implementation project to expand access to self-administered depot medroxyprogesterone acetate (DMPA)	Journal Article	USA	Safety-net family planning program involving women in an urban, primary care setting	3, 4
3	Bertrand et al. 2018 [27]	An observational study to test the acceptability and feasibility of using medical and nursing students to instruct clients in DMPA-SC self-injection at the community level in Kinshasa	Journal Article	Democratic Republic of Congo (DRC)	DMPA SC self-injection community outreach program using ﻿medical and nursing students to instruct clients	1, 3, 4
4	PATH; JSI Inc. 2021 [28]	Building capacity through digital approaches: Can eLearning replace in-person training? – DMPA-SC Resource Library	Report	Senegal and Uganda	DMPA-SC Access Collaborative facilitated presentations and reports for DMPA-SC self-injection projects involving multiple stakeholders, including women, health workers, policymakers, implementing partners, non-governmental organizations, and ministries of health	1, 4
5	PATH; JSI Inc. 2021 [29]	Costing and funding analysis for DMPA-SC program planning – DMPA-SC Resource Library	Report	Not specified	DMPA-SC Access Collaborative facilitated presentations and reports for DMPA-SC self-injection projects involving multiple stakeholders, including women, health workers, policymakers, implementing partners, non-governmental organizations, and ministries of health	2, 4
6	PATH; JSI Inc. 2021 [30]	Counting on the Private Sector to Understand the Total Market: Considerations for DMPA-SC data collection, reporting, and use – DMPA-SC Resource Library	Report	Benin, Burkina Faso, Guinea, Kenya, Madagascar, Malawi, Mali, Myanmar, Nigeria, Togo, Uganda, and Zambia	DMPA-SC Access Collaborative facilitated presentations and reports for DMPA-SC self-injection projects involving multiple stakeholders, including women, health workers, policymakers, implementing partners, non-governmental organizations, and ministries of health	1, 2, 3, 4
7	PATH; JSI Inc. 2021 [9]	Democratic Republic of the Congo's journey to DMPA-SC and self-injection scale-up – DMPA-SC Access Collaborative Country Briefs	Report	Democratic Republic of Congo	DMPA-SC Access Collaborative country brief describing self-injection scale-up	1, 3, 4
8	PATH; Kenya Ministry of Health 2018 [31]	DMPA-SC Evidence to Practice Meeting: Increasing Access, Empowering Women Meeting Report: Nairobi, Kenya – DMPA-SC Evidence to Practice Meeting Reports	Report	Bangladesh, Benin, Burkina Faso, Cote d'Ivoire, DRC, Ghana, India, Kenya, Madagascar, Malawi, Mali, Mozambique, Myanmar, Niger, Nigeria, Senegal, Uganda, and Zambia.	DMPA-SC Access Collaborative facilitated presentations and reports for DMPA-SC self-injection projects involving multiple stakeholders, including women, health workers, policymakers, implementing partners, non-governmental organizations, and ministries of health	1, 2, 3, 4
9	Health Policy Plus (HP+) 2018 [32]	DMPA-SC Introduction and Scale-Up in Nigeria: Future Benefits for Contraceptive Use and Savings – Health Policy Plus (HP+) Policy Brief	Report	Nigeria	A collaborative project between implementing partners and Nigeria's Federal Ministry of Health to quantify the impact and cost implications of DMPA-SC introduction and scale-up in Nigeria	1, 2, 3, 4
10	PATH; JSI Inc. 2021 [3]	Expanding Access to Contraception through Global Collaboration – DMPA-SC Resource Library	Report	Global report	Public, private, and philanthropic organizations global support program to scale up of DMPA-SC in FP2020 countries	1, 2, 3, 4
11	Reproductive Health Access Project 2020 [33]	Expanding Access to the Self-Administered Contraceptive Injection	Report	USA	Multi-stakeholder advocacy project for DMPA-SC self-administration, insurance coverage, and pharmacy availability	3, 4
12	PATH; JSI Inc. 2021 [34]	Getting the most out of HMIS data on contraceptive self-injection – DMPA-SC Resource Library	Report	Nigeria, Senegal, and Uganda	DMPA-SC Access Collaborative facilitated presentations and reports for DMPA-SC self-injection projects involving multiple stakeholders, including women, health workers, policymakers, implementing partners, non-governmental organizations, and ministries of health	1, 4
13	PATH; JSI Inc. 2021 [4]	How self-injection contributes to contraceptive autonomy and the power of making self-injection count – DMPA-SC Resource Library	Report	Global report	DMPA-SC Access Collaborative facilitated presentations and reports for DMPA-SC self-injection projects involving multiple stakeholders, including women, health workers, policymakers, implementing partners, non-governmental organizations, and ministries of health	3, 4
14	PATH, JSI Inc. et al. 2021 [35]	Integrating self-care methods into the National Health Information System: Experiences and lessons learned from Malawi – DMPA-SC Resource Library	Report	Malawi	DMPA-SC Access Collaborative facilitated presentations and reports for DMPA-SC self-injection projects involving multiple stakeholders, including women, health workers, policymakers, implementing partners, non-governmental organizations, and ministries of health	1, 3, 4
15	PATH; JSI Inc.2021 [36]	Interim and complementary data solutions – DMPA-SC Resource Library	Report	Bangladesh, Benin, Burkina Faso, Cote d'Ivoire, DRC, Ghana, India, Kenya, Madagascar, Malawi, Mali, Mozambique, Myanmar, Niger, Nigeria, Senegal, South Sudan, Uganda, and Zambia.	DMPA-SC Access Collaborative facilitated presentations and reports for DMPA-SC self-injection projects involving multiple stakeholders, including women, health workers, policymakers, implementing partners, non-governmental organizations, and ministries of health	1, 2, 3, 4
16	PATH; JSI Inc. 2021 [8]	Kenya's journey to DMPA-SC and self-injection scale-up – DMPA-SC Access Collaborative Country Briefs	Report	Kenya	DMPA-SC Access Collaborative country brief describing self-injection scale-up	1, 2, 3, 4
17	PATH; JSI Inc. 2021 [37]	Launching the family planning data toolkit for DMPA-SC self-injection	Report	Not specified	DMPA-SC Access Collaborative facilitated presentations and reports for DMPA-SC self-injection projects involving multiple stakeholders, including women, health workers, policymakers, implementing partners, non-governmental organizations, and ministries of health	4
18	PATH; JSI Inc. 2021 [38]	Looking back, thinking forward, and scaling up: Insights from the DMPA-SC Access Collaborative – DMPA-SC Resource Library	Report	Nigeria, Uganda, Madagascar, Zambia, Senegal	DMPA-SC Access Collaborative facilitated presentations and reports for DMPA-SC self-injection projects involving multiple stakeholders, including women, health workers, policymakers, implementing partners, non-governmental organizations, and ministries of health	1, 2, 3, 4
19	PATH; JSI Inc. 2021 [12]	Madagascar's journey to DMPA-SC and self-injection scale-up – DMPA-SC Access Collaborative Country Briefs	Report	Madagascar	DMPA-SC Access Collaborative country brief describing self-injection scale-up	1, 2, 3, 4
20	Pathfinder International 2021 [39]	Monitoring of National DMPA-SC Scale-up GAT.2218-01665802	Report	Democratic Republic of Congo	A country brief describing self-injection scale-up by Pathfinder International	1, 2, 3, 4
21	PATH; JSI Inc. 2021 [11]	Nigeria's journey to DMPA-SC and self-injection scale-up – DMPA-SC Access Collaborative Country Briefs	Report	Nigeria	DMPA-SC Access Collaborative country brief describing self-injection scale-up	1, 2, 3, 4
22	Osinowo et al. 2021 [40]	Resilient and Accelerated Scale-Up of Subcutaneously Administered Depot–Medroxyprogesterone Acetate in Nigeria (RASuDiN): A Mid-Line Study in COVID-19 Era	Journal Article	Nigeria	A training program for healthcare service providers and community-oriented resource providers on DMPA-SC	1, 3, 4
23	Evidence to Action (E2A) Project 2021 [41]	Scaling-Up Community-Based Counselling and Distribution of DMPA-SC in the DRC – DRC TECHNICAL BRIEF	Report	Democratic Republic of Congo	Community-based family planning projects involving women, health workers, implementers, and policymakers	1, 2, 3, 4
24	PATH; JSI Inc. et al. 2020 [42]	Second DMPA-SC Evidence to Practice Meeting: Increasing Access, Empowering Women: Meeting Report: Dakar, Senegal – DMPA-SC Evidence to Practice Meeting Reports	Report	Benin, Burkina Faso, Côte d'Ivoire, the Democratic Republic of the Congo, Ghana, Guinea, Kenya, Madagascar, Malawi, Mali, Mauritania, Mozambique, Myanmar, Niger, Nigeria, Pakistan, Senegal, Togo, Uganda and Zambia	Meeting report involving country delegations, donor organizations, bilateral and multilateral organizations, and non-governmental organizations	1, 2, 3, 4
25	Hernandez et al. 2018 [43]	Task-shifting the provision of DMPA-SC in the DR Congo: Perspectives from two different groups of providers	Journal Article	Democratic Republic of Congo	DMPA-SC self-administration implementation program involving training and supervision of medical and nursing school students and lay community health workers	1, 3, 4
26	PATH; CHAI et al. 2021 [44]	The Catalytic Opportunity Fund for Scale-Up of DMPA-SC: Learning from high-impact, short-term funding opportunities – DMPA-SC Resource Library	Report	DRC, Nigeria, Kenya, Burkina Faso, Guinea, Mali, and Togo	DMPA-SC Access Collaborative facilitated presentations and reports for DMPA-SC self-injection projects involving multiple stakeholders, including women, health workers, policymakers, implementing partners, non-governmental organizations, and ministries of health	1, 2, 3, 4
27	PATH; JSI Inc. 2019 [45]	The Future of DMPA-SC: Expanding access and options in 2019 – DMPA-SC Resource Library**	Report	DRC, Ghana, Kenya, Madagascar, Malawi, Mozambique, Myanmar, Nigeria, Rwanda, Senegal, Tanzania, Uganda, Zambia, Zimbabwe	Meeting report involving country delegations, donor organizations, bilateral and multilateral organizations, and non-governmental organizations	1, 2, 4
28	Uzma et al. 2021 [46]	The role of partners in promoting self-care for misoprostol and subcutaneous DMPA in Pakistan	Journal Article	Pakistan	Country experience introducing and scaling up self-care interventions by collaboration between the ministry of health and implementing partners	1, 3, 4
29	Ntabona et al. 2021 [47]	The scale-up and integration of contraceptive service delivery into nursing school training in the Democratic Republic of the Congo	Journal Article	Democratic Republic of Congo	DMPA-SC self-administration implementation program involving nursing school students	1, 2, 3, 4
30	PATH; JSI Inc. 2021[48]	Toolkit for DMPA-SC monitoring, learning, and evaluation – DMPA-SC Resource Library	Report	Not specified	DMPA-SC Access Collaborative facilitated presentations and reports for DMPA-SC self-injection projects involving multiple stakeholders, including women, health workers, policymakers, implementing partners, non-governmental organizations, and ministries of health	4
31	PATH; JSI Inc. 2021[13]	Uganda's journey to DMPA-SC and self-injection scale-up – DMPA-SC Access Collaborative Country Briefs	Report	Uganda	DMPA-SC Access Collaborative country brief describing self-injection scale-up	1, 2, 3, 4
32	PATH; JSI Inc. et al. 2021 [49]	Unlocking DMPA-SC data-sharing between private pharmacies and ministries of health – DMPA-SC Resource Library	Report	Kenya and Zambia	DMPA-SC Access Collaborative facilitated presentations and reports for DMPA-SC self-injection projects involving multiple stakeholders, including women, health workers, policymakers, implementing partners, non-governmental organizations, and ministries of health	1, 4
33	PATH; JSI Inc.2021 [50]	What we learned and where we go from here: Making Self-Injection Count workshop – DMPA-SC Resource Library	Report	Uganda, Madagascar, DRC, Nigeria, Zambia, Benin, Burkina Faso, Cote d'Ivoire, Guinea, Mali, Mauritania, Niger, and Togo	DMPA-SC Access Collaborative facilitated presentations and reports for DMPA-SC self-injection projects involving multiple stakeholders, including women, health workers, policymakers, implementing partners, non-governmental organizations, and ministries of health	3, 4
34	PATH; JSI Inc. 2021 [10]	Zambia's journey to DMPA-SC and self-injection scale-up – DMPA-SC Access Collaborative Country Briefs	Report	Zambia	DMPA-SC Access Collaborative country brief describing self-injection scale-up	1, 2, 3, 4

*EPOC intervention domain: 1) delivery arrangements, 2) financial arrangements, 3) governance arrangements, and 4) implementation strategies

** International Conference on Family Planning Kigali, Rwanda Pre-conference Meeting Report

### Delivery arrangements

**Fig. 2:**
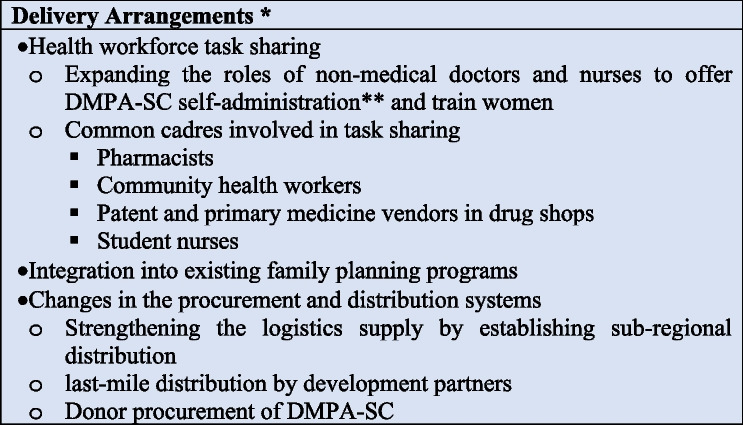
Delivery arrangements to scaling up DMPA-SC self-administration programs *How, when, and where DMPA-SC client self-administration was organized and delivered, and who delivered DMPA-SC client self-administration activities, **Self-administration of depot medroxyprogesterone acetate subcutaneous injectable contraception

Figure 2 summarizes the interventions in the EPOC framework delivery arrangements’ category. Twenty-seven documents reported at least one intervention in the delivery arrangement domain. This review identified interventions in six subcategories of delivery arrangements. First, 20 documents reported changes in the DMPA-Sc self-administration service delivery site [8–13, 25, 27, 30–32, 38–44, 47, 49]. All 20 documents reported community distribution as the site of service delivery, and of these, two were through community campaigns [43, 47]. Additionally, 18 documents had evidence of active private sector engagement (pharmacies or drug shops) [8, 10–13, 25, 28, 30–32, 36, 38–40, 42, 44, 45, 49] or employed a total market approach (*n*= 9) [8, 10, 30, 31, 38, 40, 42, 45, 49], to organize the public and private sectors as a strategy to scale up the self-administration of DMPA-SC.

Second, 15 documents reported changes in the procurement and distribution systems for DMPA-SC self-administration [9–13, 31, 32, 36, 38–41, 44, 45, 47]. The most widely reported intervention for strengthening DMPA-SC procurement and distribution for client self-administration was funding provider distribution of DMPA-SC (*n*= 6) [11–13, 38, 41, 47]. Other methods for strengthening the logistics supply for DMPA-SC were the establishment of sub-regional distribution hubs or organizations (*n*= 5) [11, 13, 32, 38, 40], last-mile distribution by development partners (*n*= 2) [10, 40], and donor procurement of DMPA-SC (*n*= 1) [45].

Third, most documents (*n*= 23) reported task sharing to expand the roles of non-medical doctors and nurses in offering and training women in the self-administration of DMPA-SC [3, 8–13, 25, 27, 28, 30, 32, 36, 38–44, 46, 47, 49]. The most common cadres involved in task sharing were pharmacists (*n*= 15) [8, 10–13, 25, 28, 30, 32, 38–40, 42, 46, 49]. Other cadres were community health workers (*n*= 10) [10–12, 25, 32, 40–43, 46], patents and primary medicine vendors (PPMVs) in drug shops (*n*= 10) [11–13, 28, 30, 32, 40, 42, 44], and student nurses (*n* = 4) [27, 41, 43, 47]. Regarding interventions in other subcategories of delivery arrangements, half of the documents in this review reported the integration of DMPA-SC self-administration into existing family planning programs to scale up the uptake of the method [9, 11–13, 25, 31, 32, 34–36, 38–42, 47, 49].

### Financial arrangements

**Fig. 3:**
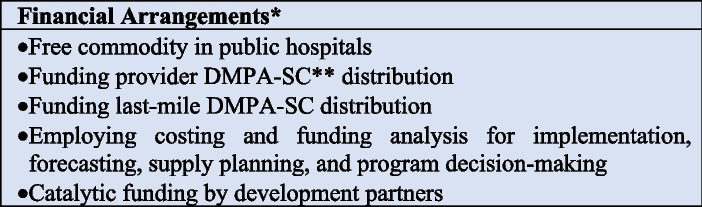
Financial arrangements to scaling up DMPA-SC self-administration programs *Insurance schemes, how funds were collected, how services were purchased, and the use of targeted financial incentives or disincentives for DMPA-SC client self-administration, **Self-administration of depot medroxyprogesterone acetate subcutaneous injectable contraception

Figure 3 summarizes the interventions in the EPOC framework financial arrangements’ category. Half of the documents in this review (*n*= 18) reported themes regarding financial arrangements [3, 8, 10–13, 30–32, 36, 38, 39, 41, 42, 44, 45, 47]. Three documents showed that DMPA-SC self-administration services were offered at no cost to clients [10, 13, 41]. None of the documents reported free DMPA-SC in the private sector. To scale up DMPA-SC self-administration, six documents reported funding provider distribution [11–13, 38, 41, 47], while one document reported funding last-mile distribution [10]. The most widely employed financial arrangement to scale up DMPA-SC self-administration was costing and funding analysis for implementation, forecasting, supply planning, and program decision-making (*n*= 8) [8, 12, 29, 32, 36, 39, 42, 45]. In five documents [3, 8, 11, 39, 44], the source of funding for activities to scale up DMPA-SC self-administration was the catalytic funds administered by the Clinton Health Access Initiative (CHAI).

### Governance arrangements

**Fig. 4:**
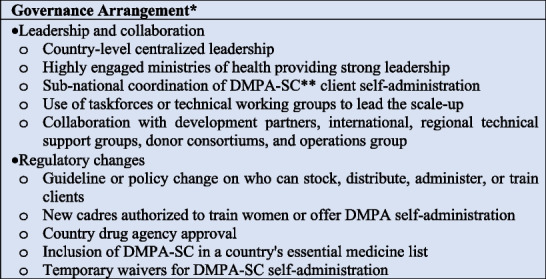
Governance arrangements to scaling up DMPA-SC self-administration programs *Rules or processes that affect how powers were exercised, particularly regarding authority, accountability, openness, participation, and coherence of DMPA-SC client self-administration programs, **Self-administration of depot medroxyprogesterone acetate subcutaneous injectable contraception

Figure 4 summarizes the interventions in the EPOC framework governance arrangements category. There were 27 documents that reported at least one intervention in the domain of governance arrangements. Almost two-thirds of the documents in this review reported country-level centralized leadership for scaling up DMPA-SC self-administration (*n*= 22) [3, 8–13, 25, 27, 30–32, 35, 36, 38–43, 46, 47]. Additionally, five documents reported sub-national coordination of the DMPA-SC self-administration [11, 13, 32, 38, 40]. Country ministries of health were highly engaged in providing strong leadership in most programs (*n*= 22) [3, 8–13, 25, 27, 30–32, 35, 36, 38–43, 46, 47]. In 10 documents, taskforces or technical working groups were set up to lead the scale-up of DMPA-SC self-administration [10–13, 30, 35, 36, 38, 39, 47]. Over two-thirds of the documents (*n*= 23) reported collaboration with development partners or cross-sectional collaboration beyond the health ministry or collaboration with international regional technical support groups or donor consortium and operations group or all to scale up DMPA-SC self-administration programs [3, 4, 8–13, 25, 27, 31, 32, 36, 38–44, 46, 47, 50].

The most prominent regulatory change affecting self-administration of DMPA-SC is a guideline or policy change on who can stock, distribute, administer, or train clients on the use of the commodity (*n*= 17) [3, 8–13, 25, 30, 32, 36, 38–42, 47]. New cadres authorized to train women or offer DMPA self-administration to women were student nurses (*n*= 4) [27, 41, 43, 47], pharmacists (*n*= 10) [8, 10–13, 30, 32, 39, 40, 42], and drug store sellers (*n* = 8) [11–13, 30, 32, 39, 40, 42]. Other regulatory changes to scale up DMPA-SC self-administration included country drug agency approval for DMPA-SC self-administration (*n*= 12) [3, 8, 11–13, 25, 32, 35, 36, 39, 40, 42], and adding DMPA-SC in a country’s essential medicine list (*n*= 7) [8, 11–13, 36, 39, 42]. In two documents, temporary waivers were granted for DMPA-SC self-administration [26, 33].

### Implementation strategies

**Fig. 5:**
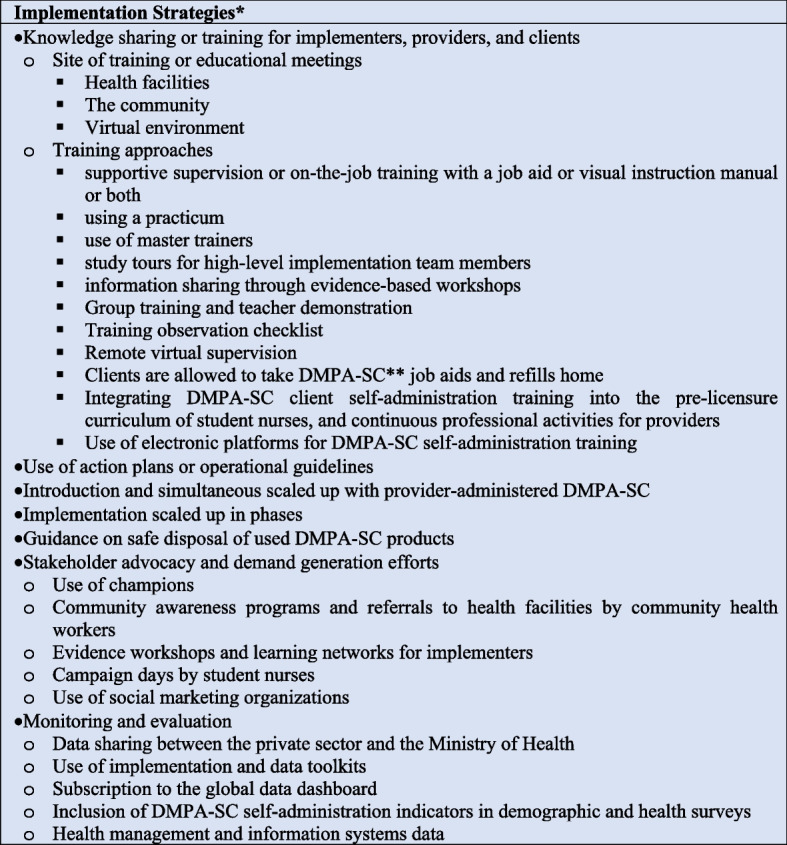
Implementation strategies to scaling up DMPA-SC self-administration programs *Interventions designed to bring about changes in healthcare organizations, the behavior of healthcare professionals, and the use of health services by healthcare recipients **Self-administration of depot medroxyprogesterone acetate subcutaneous injectable contraception

Figure 5 summarizes the interventions in the EPOC framework implementation strategies’ category. All documents in this review had at least one intervention in the implementation strategy domain. The most widely reported implementation strategy was knowledge sharing or training for implementers, providers, and clients (*n*= 30) [3, 8, 11–13, 25–32, 35–46, 48, 49]. Training or educational meetings were conducted in health facilities (*n*= 19) [3, 9–13, 25, 26, 28, 30, 32, 38–44, 47], the community (*n* = 17) [8, 9, 11, 12, 25, 27, 30, 32, 38–41, 43, 44, 46, 47, 49], or via the virtual environment (*n*= 6) [3, 8, 13, 28, 36, 38]. The most widely used approach to training was supportive supervision or on-the-job training with a job aid or visual instruction manual or both (*n*= 16) [3, 10–13, 25, 28, 36, 38–40, 43, 44, 46, 47]. After training, some programs allowed clients to take DMPA-SC refills (*n*= 2) [25, 38], and job aids home (*n*= 2) [13, 38]. Other approaches were training using a practicum (*n*= 3) [28, 43, 47], use of master trainers (*n*= 8) [8, 9, 11, 25, 40, 41, 44, 47], study tours for high-level implementation team members (*n*= 9) [8–13, 31, 36, 39], information sharing through evidence-based workshops (*n*= 3) [31, 39, 42], group training and provider demonstration (*n*= 3) [13, 38, 42], training observation checklist (*n* = 2) [37, 43], and remote virtual supervision (*n*= 3) [3, 36, 38]. Additionally, five documents each reported on the use of electronic platforms for DMPA-SC self-administration training [3, 8, 13, 28, 38]. Some documents also show that DMPA-SC self-administration training was incorporated into the pre-licensure curriculum of student nurses [47], and family planning training curriculum for health care providers, pharmacists, pharmaceutical technologists, community-based distributors, and community health workers [8, 9, 12].

More than half of the documents in this scoping review reported that DMPA-SC self-administration scale-up programs had action plans or operational guidelines (*n*= 20) [8–13, 25, 30–32, 35, 36, 38–42, 45, 47, 50]. In five documents, the operational guidelines included client journey maps. This tool showed how a client accessed DMPA-SC and performed self-injection [10, 12, 13, 30, 42]. Three documents reported that self-administration of DMPA-SC was introduced and scaled up simultaneously with provider-administered DMPA-SC [11, 12, 25], and one document reported that self-injection was scaled up in phases [47]. Of the documents that reported operational guidelines for DMPA-SC self-administration, four reported the availability of guidance on safe disposal of used DMPA-SC products.

Other implementation strategies to scale up DMPA-SC in this review were stakeholder advocacy and demand generation efforts (*n*= 26) [3, 8–13, 25–27, 30–36, 38–44, 46, 47, 49], as well as interventions for monitoring and evaluation (*n*= 23) [3, 4, 8, 9, 11–13, 30, 34–44, 47–50]. Examples of stakeholder advocacy and demand generation efforts were as follows: use of champions (*n*= 3) [10, 13, 38], community awareness programs and referrals to health facilities by community health workers (*n*= 3) [10, 32, 43], evidence workshops and learning networks for implementers (*n*= 3) [31, 39, 42], campaign days by student nurses (*n*= 2) [43, 47], and the use of social marketing organizations (*n*= 4) [3, 32, 39, 40]. The monitoring and evaluation interventions reported were as follows: data sharing between the private sector and the Ministry of Health (*n*= 5) [36, 38–40, 49], use of implementation and data toolkits (*n*= 6) [3, 36–38, 43, 48], subscriptions to the global data dashboard (*n*= 5) [4, 38, 39, 42, 50], and adding DMPA-SC self-administration indicators in demographic and health surveys (DHS) [4, 36], and health management and information systems data (*n*= 16) [3, 4, 8, 9, 11–13, 28, 34–36, 38–40, 42, 47, 50].

### Implementation barriers

**Fig. 6:**
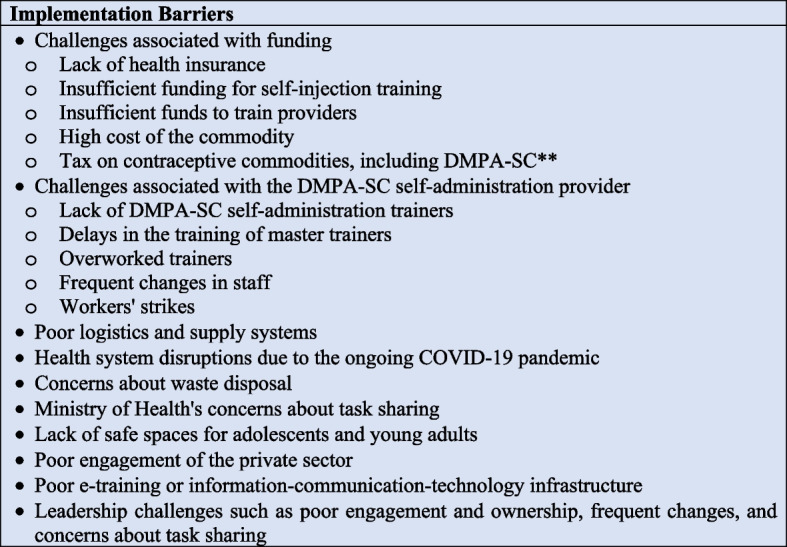
Implementation barriers to scaling up DMPA-SC self-administration programs **Self-administration of depot medroxyprogesterone acetate subcutaneous injectable contraception

Figure 6 summarizes the barriers to scaling up DMPA-SC client self-administration programs. Over two-thirds of the documents (*n*= 23) had at least one barrier to scaling up DMPA-SC self-administration programs [3, 8–13, 25–28, 33, 34, 36, 38–42, 44–47]. The most common barriers were challenges associated with financing DMPA-SC self-administration programs, such as lack of health insurance, insufficient funding for self-injection training, and high cost of the commodity (*n*= 16) [8, 11–13, 26–28, 33, 34, 38, 39, 41, 42, 45–47]. In 14 documents, challenges were associated with the unavailability and performance of DMPA-SC providers proficient in offering self-administration training to other providers or clients. These challenges were due to a lack of DMPA-SC self-administration trainers, delays in the training of master trainers, overworked trainers, frequent changes in staff, workers’ strikes, and insufficient funds to train providers [11–13, 28, 34, 38–42, 44–47].

Almost half of the documents (*n*= 15) identified poor logistics and supply systems as barriers to scaling up DMPA-SC self-administration [8–11, 13, 25, 27, 36, 39–42, 45–47]. In 14 documents, health system disruptions due to the ongoing COVID-19 pandemic was cited as a barrier to scale-up programs [3, 8, 9, 11–13, 26, 28, 33, 36, 39–41, 47]. Other barriers were as follows: concerns about waste disposal (*n*= 7) [13, 25, 27, 38, 39, 42, 45], Ministry of Health’s concerns about the ability of community health workers to offer self-administration services (*n*= 3) [12, 39, 42], tax on contraceptive commodities including DMPA-SC (*n*= 3) [11, 12, 42], lack of safe spaces for adolescents and young adults (*n*= 2) [13, 40], poor engagement of the private sector (*n*= 5) [12, 26, 33, 39, 42], poor e-training or information-communication-technology infrastructure (*n*= 4)[28, 36, 40, 47], and leadership challenges such as poor engagement and ownership and frequent changes (*n* = 6) [8, 9, 39, 40, 42, 47].

### Intervention outcomes

All the scale-up interventions for DMPA-SC self-administration included in this review had at least some indirect evidence in the source documents that they were feasible to implement. However, there was limited information on fidelity, i.e., the degree of intervention implementation as intended in an ideal situation. Regarding penetration, “the integration of a practice within a service setting and its subsystems,” [22] half of the documents in this review reported the integration of DMPA-SC self-administration services into family planning programs [9, 11–13, 25, 26, 32, 34–36, 38–42, 47, 49].

A DMPA-SC Consortium report showed that at least 20 of 31 countries introducing or scaling up DMPA-SC are planning for or introducing self-injection [3]. The report also showed that the proportion of DMPA-SC users that opted for self-administration in Malawi, Uganda, and Togo were 44, 30, and 29%, respectively [3]. However other locations had a lower proportion of self-administration, i.e., 5–10% [3]. In Madagascar, Uganda, and Malawi, the proportion of DMPA-SC used for self-administration were 3, 13, and up to 25% (per month), respectively [36]. Report shows that Zambia [10], Madagascar [12] Uganda [13], and Togo [3] offer DMPA-SC self-administration in 25, 49, and 41%, and all public health service delivery points.The Democratic Republic of Congo (DRC) [9] offers self-administration in 47% of their health zones. At the evidence to practice meeting in Kenya [31], reports from Madagascar noted that scale-up efforts were associated with increased satisfaction among users. The Ministry of Health was satisfied with the user rates and DMPA-SC logistics and supply compared with intra-muscular DMPA.

Multi-country reports by the DMPA Access Collaborative show that about 80,000 providers were trained in DMPA-SC self-administration between 2018 and 2020 [3, 4]. Using reported and modeled data, the proportion of DMPA visits for self-administration by country was 10–45% [4]. Nigeria [11], the DRC [9], Madagascar [12], and Zambia [10] have trained 28,200, 7400, 3600, and 3100 DMPA-SC providers constituting 21 and 13% of DRC and Madagascar targets, respectively. The “Self-Injection Best Practices (SIBP) project,” a flagship user-centered training led by PATH, trained about 13,000 women on DMPA-SC self-administration in Uganda [13]. Of these, more than 7000 women had started using the method. A document reported improved knowledge acquisition after a training exercise [8], another noted an increased completion rate of modules when using electronic learning platforms [28], and the third reached more providers via virtual supervision [38].

A report on the catalytic funds administered by the Clinton Health Access Initiative (CHAI) showed that it supports DMPA-SC services in 14 countries [44]. Regarding financial interventions, the catalytic funds had supported the training of over 300, 10,000, 2000, and 9000 master trainers, public providers, private providers, and clients on DMPA-SC self-administration, respectively [44]. Further, of 20 countries at the second “Evidence to Practice Meeting” in Senegal, 16 had costed plans for their DMPA programs [42]. However, only 3 of the 13 countries have met at least half of their 2018 and 2019 target [42]. A report from Nigeria showed that though introduction and scaling up would cost about US$80 million, a return on investment of US$49 million is expected over 5 years [32].

## Discussion

### Main findings

This scoping review identified 34 documents reporting interventions to scale up DMPA-SC self-administration uptake globally and the barriers and implementation outcomes of the programs. Most documents reported projects or country-level programs in Africa between 2018 and 2021. We noted a relative lack of peer-reviewed articles reporting scaled up strategies for DMPA self-administration and the direct implementation outcomes of such programs. Using the EPOC taxonomy of health systems framework [24], all documents had at least one intervention in the implementation strategy domain (*n* = 34). The contributions of the other domains were *n* = 27, *n* = 27, and *n* = 18 documents on healthcare delivery, governance, and financial arrangements, respectively. Despite having the lowest proportion of documents in the financial arrangements domain, challenges associated with funding were the most-reported barriers to scaling up DMPA-SC self-administration programs. Few documents reported outcomes directly linked to scaled up interventions in this review.

### Strengths and limitations

To our knowledge, this is the first review to report on interventions to scale up DMPA-SC self-administration. This scoping review search strategy was exhaustive, with six databases and a scheme to explore the grey literature. The search strategy followed the most recent guidelines for conducting and reporting systematic searches [16–18] and was peer-reviewed using the Peer Review of Electronic Search Strategies (PRESS) guideline [23]. To cater to documents not in searchable archives, we requested relevant documents from online networks and listservs for people implementing programs on DMPA-SC. The narrative approach to presenting evidence in this review also permitted a more meaningful synthesis of qualitative and quantitative data from the included documents.

Although we noted some overlap between the main categories of the EPOC taxonomy, this study utilized the EPOC framework to structure the interventions identified into 4 domains [24]. For instance, some overlap existed between (1) the governance arrangements that permitted task sharing between health cadres and (2) task sharing as a delivery arrangement to scale up DMPA-SC self-administration programs. We presented information in the two domains as appropriate. The EPOC framework allows for an easier understanding and comparison between similar studies and programs. Furthermore, we noted a poor distinction between the interventions and facilitators of scaled up programs in the documents in this review. Hence, we decided to analyze only interventions to scale up programs and barriers to such interventions without further mapping facilitators and barriers using the capability, opportunity, and motivation behavior system as planned [51].

Although this review utilized a robust methodology for the grey literature search, curating all sources was impossible. Furthermore, not all documents included in this review were set out to report the interventions, facilitators, barriers, and implementation outcomes of programs to scale up DMPA-SC self-administration. Thus, the mode of information presented in some documents was not explicit. We ensured that the extracted data were reasonably related to details in all the documents. Although this review summarized the number of documents reporting an intervention to scale-up DMPA-SC self-administration by document counting, a higher number does not translate to a better intervention because it is not a test of statistical associations, and documents in this review may include duplicated data. Because of the varied types of documents contained in this scoping review and in line with the methodological guidance of Arksey and O'Malley [3], the quality of the included studies was not assessed. To synthesize our results, we employed a narrative approach applicable to scoping review methodology. Therefore, the strength of the evidence summarized in this review is not absolute.

### Interpretation and suggestions for future research

Similar to other reviews that reported on interventions to scale up health programs [52–57], this review identified the following notable interventions: task-sharing amongst health workforce cadres, engaged leadership, encouraging policies, training and education, DMPA-SC demand generation, integration into existing programs, improved funding mechanisms, collaboration with development partners, and supply chain strengthening. The main barriers to scaling up DMPA-SC self-administration were suboptimal funding, an insufficient number of trained health personnel to offer services, and poor logistics and supply systems. These barriers have also been reported in reviews assessing the scale-up of other health programs [55, 58, 59].

Although frameworks exist to aid the structured presentation of implementation outcomes [22], only a few documents in this review had a structured approach to reporting scale-up outcomes. While many women opted for self-administration of DMPA-SC across different program and countries, the review could not ascertain the best-performing projects because of wide-ranging project designs and performance indicators [3, 36]. For instance, while some programs reported the proportion of women that opted for DMPA-SC self-administration, or proportion of facilities offering DMPA-SC self-administration, others noted the proportion of DMPA-SC used for self-administration [3, 9, 10, 12, 13, 36].

There was a paucity of documents that reported scale-up outcomes possibly because documenting sustainability (an element of scale-up) requires lengthier program monitoring [60], or because existing programs placed a limited premium on reporting the impact of scale-up interventions compared to documenting the process of scale-up or outputs of interventions implemented. Unlike the results of pilot studies that showed more continuation rates with DMPA SC self-administration [61], the evidence regarding the effect of implementation strategies, facilitators and barriers on continuing rates was scanty in this review. This finding is similar to other studies, which reported little evidence on scale up (i.e., penetration and sustainability) [60, 62, 63]. Consequently, this review was unable to present a structured analysis of the outcomes of DMPA-SC self-administration programs using the Proctor et al. framework [22].

This scoping review aimed to describe the interventions to scale up self-administered DMPA-SC programs, the barriers and facilitators of these programs, and the outcomes of the implementation strategies. Our research shows that most of the evidence in this review was descriptive summaries of interventions to scale up DMPA self-administration. Thus, there is a need for well-conducted implementation research that rigorously assess scaled up interventions and documents their outcomes. Since most programms or countries are likely to utilize a package of interventions to scale up DMPA self-administration, it is essential to determine how each component is selected and the required level of implementation of each component to yield a desired outcome.

## Conclusion

Overall, this scoping review reported a wide range of interventions employed by countries and programs to scale up DMPA-SC self-administration and the barriers encountered. The most-reported interventions were: task-sharing amongst health workforce cadres, engaged leadership, encouraging policies, training and education, DMPA-SC demand generation, integration into existing programs, improved funding mechanisms, collaboration with development partners, and supply chain strengthening. At the same time, the main barriers were suboptimal funding, inadequate human resources, and poor logistics supply of DMPA-SC. There is minimal evidence of the implementation outcomes. Going for-ward, efforts should focus on rigorous implementation research that assess scaled up DMPA-SC self-administration interventions and reports their outcomes.

## Supplementary Information

**Additional file 1: Supplementary material.** Search strategies.

## Data Availability

All data generated and analyzed are available in the manuscript and the supplementary materials.

## Abbreviations

DMPA-SC: Depot medroxyprogesterone acetate subcutaneous injectable contraception
EPOC: Effective Practice and Organization of Care
PRESS: Peer Review of Electronic Search Strategies
PRISMA: Preferred Reporting Items for Systematic Reviews and Meta-Analyses
SDG: Sustainable Development Goals
WHO: World Health Organization
