# Supplementary material for: Implementation strategies to scale up self-administered depot medroxyprogesterone acetate subcutaneous injectable contraception: a scoping review
Source: Syst Rev. 2023 Jul 4;12:114. doi: 10.1186/s13643-023-02216-2 (PMC10318699; doi:10.1186/s13643-023-02216-2)
Supplement: Supplementary file 1 — Additional file 1: Supplementary material. Search strategies. [file 13643_2023_2216_MOESM1_ESM.docx]

**7.0 SUPPLEMENTARY MATERIALS - SEARCH STRATEGIES**

**7.1 Database 1: Embase 1974 to present**

**Search: 12/10/2021**

--------------------------------------------------------------------------------

1. ((Depo-Provera or Sayana-Press or depot-medroxyprogesterone-acetate or depo-medroxyprogesterone-acetate or Depo-Medroxyprogesterone-Acetate or Medroxyprogesterone or Medroxyprogesterone-Acetate or DMPA or DMPA-SC or Uniject or Depo-Subq-Provera).ti,ab.) or medroxyprogesterone-acetate/ or medroxyprogesterone/ or long-acting-reversible-contraception/ 23921
2. ((self-administ* or self-inject* or self-management* or self-care or self-assessment* or self-treatment* or patient-management* or home-use or home-administ* or home-inject* or self-vs-provider-administ* or self-and-provider-administ* or self-vs-physician-administ* or self-and-physician-administ* or self-and-clinic* or self-vs-clinic*).ti,ab.) or drug-self-administration/ or self-care/ or Self Medication/ or self injection/ 213636
3. 1 and 2 = 179

**7.2 Database 2: Medline (Ovid MEDLINE® Epub Ahead of Print, In-Process & Other Non-Indexed Citations, Ovid MEDLINE® Daily and Ovid MEDLINE®) 1946 to present**

**Search: 12/10/2021**

--------------------------------------------------------------------------------

1. ((Depo-Provera or Sayana-Press or depot-medroxyprogesterone-acetate or depo-medroxyprogesterone-acetate or Depo-Medroxyprogesterone-Acetate or Medroxyprogesterone or Medroxyprogesterone-Acetate or DMPA or DMPA-SC or Uniject or Depo-Subq-Provera).ti,ab.) or Medroxyprogesterone-Acetate/ or Medroxyprogesterone/ or Long-Acting Reversible Contraception/ 10260
2. ((self-administ* or self-inject* or self-management* or self-care or self-assessment* or self-treatment* or patient-management* or home-use or home-administ* or home-inject* or self-vs-provider-administ* or self-and-provider-administ* or self-vs-physician-administ* or self-and-physician-administ* or self-and-clinic* or self-vs-clinic*).ti,ab.) or Self Administration/ or Self-Management/ or Self Medication/ 136008
3. 1 and 2 = 75

**7.3 Database 3: EBSCOhost CINAHL**

**Search 12/10/2021**

--------------------------------------------------------------------------------

1. TI (Depo-Provera or Sayana-Press or depot-medroxyprogesterone-acetate or depo-medroxyprogesterone-acetate or Depo-Medroxyprogesterone-Acetate or Medroxyprogesterone or Medroxyprogesterone-Acetate or DMPA or DMPA-SC or Uniject or Depo-Subq-Provera) OR AB (Depo-Provera or Sayana-Press or depot-medroxyprogesterone-acetate or depo-medroxyprogesterone-acetate or Depo-Medroxyprogesterone-Acetate or Medroxyprogesterone or Medroxyprogesterone-Acetate or DMPA or DMPA-SC or Uniject or Depo-Subq-Provera) OR MH (Medroxyprogesterone) or MH (Long-Acting Reversible Contraceptives) OR MH (Long-Acting-Reversible-Contraceptives) 1815
2. TI (self-administ* or self-inject* or self-management* or self-care or self-assessment* or self-treatment* or patient-management* or home-use or home-administ* or home-inject* or self-vs-provider-administ* or self-and-provider-administ* or self-vs-physician-administ* or self-and-physician-administ* or self-and-clinic*-administ* or self-vs-clinic*) OR AB (self-administ* or self-inject* or self-management* or self-care or self-assessment* or self-treatment* or patient-management* or home-use or home-administ* or home-inject* or self-vs-provider-administ* or self-and-provider-administ* or self-vs-physician-administ* or self-and-physician-administ* or self-and-clinic* or self-vs-clinic*) OR MH (self care) OR MH (self-care) OR MH (Self-Administration) or MH (self-medication) or MH (self-management) 89196
3. 1 and 2 = 49

**7.4 Database 4: Scopus**

**Search: 12/10/2021**

--------------------------------------------------------------------------------

1. Depo-Provera or Sayana-Press or depot-medroxyprogesterone-acetate or depo-medroxyprogesterone-acetate or Depo-Medroxyprogesterone-Acetate or Medroxyprogesterone or Medroxyprogesterone-Acetate or DMPA or DMPA-SC or Uniject or Depo-Subq-Provera 25043
2. self-administ* or self-inject* or self-management* or self-care or self-assessment* or self-treatment* or patient-management* or home-use or home-administ* or home-inject* or self-vs-provider-administ* or self-and-provider-administ* or self-vs-physician-administ* or self-and-physician-administ* or self-and-clinic* or self-vs-clinic* 220672
3. 1 and 2 = 167

**7.5 Database 5: Web of science - Core Collection**

**Search: 12/10/2021**

--------------------------------------------------------------------------------

1. TS=(Depo-Provera or Sayana-Press or depot-medroxyprogesterone-acetate or depo-medroxyprogesterone-acetate or Depo-Medroxyprogesterone-Acetate or Medroxyprogesterone or Medroxyprogesterone-Acetate or DMPA or DMPA-SC or Uniject or Depo-Subq-Provera or Long-Acting-Reversible-Contracept*) 11183
2. TS=(self-administ* or self-inject* or self-management* or self-care or self-assessment* or self-treatment* or patient-management* or home-use or home-administ* or home-inject* or self-vs-provider-administ* or self-and-provider-administ* or self-vs-physician-administ* or self-and-physician-administ* or self-and-clinic* or self-vs-clinic*) 151272
3. 1 and 2 = 95

**7.6 Database 6: Google Scholar**

**Search 14/12/2021**

--------------------------------------------------------------------------------

1. related: "medroxyprogesterone AROUND (3) acetate" "self AROUND(3) administer"
2. Results = 190
